# Antibiotic synergy testing for multidrug-resistant Gram-negative pathogens in a Greek ICU

**DOI:** 10.1186/cc14177

**Published:** 2015-03-16

**Authors:** E Douka, E Perivoliot, E Kraniotaki, M Nepka, C Routsi, K Fountoulis, A Skoutelis, S Zakynthinos

**Affiliations:** 1 Evangelismos General Hospital, Athens, Greece

## Introduction

The emergence of multidrug-resistant (MDR) pathogens is a major cause of infection-related mortality among critically ill patients. The synergistic effect between commonly used antibiotics against difficult to treat nosocomial MDR Gram-negative strains, if present, could provide a viable option as an alternative therapy. The aim of this study was to investigate the potential of antibiotic synergy against MDR *A. baumannii, K. pneumonia *and *P. aeruginosa *strains, isolated from critically ill patients in a Greek ICU.

## Methods

We tested 59 *A. baumannii*, 41 *K. pneumoniae *and 64 *P. aeruginosa *strains, isolated during the period 2010 to 2013. All strains were resistant to carbapenems and showed reduced susceptibility or resistance to tigecycline or colistin (MIC >2), in accordance with CLSI guidelines. We evaluated double-drug combinations of carbapenem (CRB)/colistin (COL), tigecycline (TG)/COL, rifampicin (RIF)/COL, CRB/ gentamicin (GEN), CRB/amikacin (AMK) for *A. baumannii*, TG/COL, CRB/COL, piperacillin-tazobactam (PIP/TAZ)/GEN, CRB/GEN for *K. pneumoniae *and AMK/(PIP/TAZ), AMK/aztreonam (AZT), AMK/cefepime (CEF), AMK/CRB and CRB/COL for *P. aeruginosa *strains. In order to perform synergy tests, the E-test methodology (BioMerieux, Marcy l'E'toile, France) was used. Synergy was defined as a fraction inhibitory concentration (FIC) index ≤0.5, additive effect 0.5 to 1, indifferent or antagonistic effect >2 (Lorian definition).

## Results

Against 59 MDR *A. baumannii *strains, the synergy effect of CRB/ COL was 55.9%, RIF/COL 38.9%, CRB/GEN 22%, CRB/AMK 20.3% and TG/COL 16.9%, respectively. Against 41 *K. pneumoniae *strains, synergy rates were: CRB/COL 43.9%, CRB/GEN 31.7%, PIP/TAZ/GEN 29.2% and TG/COL 24.4% respectively. Against 64 *P. aeruginosa *strains, synergy rates were: AMK/PIP/TAZ 64.6%, AMK/AZT 64.6%, AMK/CEF 58.3%, CRB/ COL 52%, AMK/CRB 25%.

## Conclusion

The most effective combination for both the *A. baumannii *and *K. pneumoniae *strains tested was CRB/COL. The next most effective combination was RIF/COL and CRB/GEN respectively. No competitive effect was observed for RIF/COL combination in all cases tested. The most effective combinations for *P. aeruginosa *strains were AMK plus PIP/TAZ or AZT or CEF. The next most effective combination was CRB/ COL. We recommend implementation of an antibiotic synergy test for MDR pathogens as a routine antimicrobial test in the hospitals' microbiology laboratories, especially for critically ill patients, since some combinations seem to excel. Further studies are needed for the correlation of these combinations with clinical efficacy.

